# Survival and predictors of mortality among acute leukemia patients on follow‐up in Tikur Anbessa Specialized Hospital, Addis Ababa, Ethiopia: A 5‐year retrospective cohort study

**DOI:** 10.1002/cnr2.1890

**Published:** 2023-10-02

**Authors:** Bargude Balta, Tigistu Gebreyohannis, Erdaw Tachbele

**Affiliations:** ^1^ Department of Nursing Hawassa University Comprehensive Specialized Hospital Hawassa Ethiopia; ^2^ College of Health Sciences Addis Ababa University Addis Ababa Ethiopia

**Keywords:** Ethiopia, hematologic, leukemia, lymphoblastic, myeloid

## Abstract

**Background:**

Although Ethiopia has more than 78% of leukemia cases and a significant burden of the disease, the survival of leukemia patients in the country is poorly recognized. The purpose of this study was to assess the survival and predictors of acute leukemia patients.

**Methods:**

A 5‐year retrospective cohort study was conducted including all acute Leukemia patients who visited Tikur Anbessa Specialized Hospital between January 2015 and December 2019. Data were retrieved from patient's medical records between March and April 2020. Using SPSS version 25, the Kaplan–Meier curve and Cox regression models were employed to analyze the data.

**Results:**

A total of 119 patients with acute leukemia were retrospectively evaluated for 60 months, having 196 person‐years of risk. About 46 deaths (38.7%) were recorded over the follow‐up period, giving a mortality incidence rate of 23.5 (95% CL:18–52) per 100 person‐years. The median survival time was 35 months (95% CI, 28.3–41.7). At 60 months of follow‐up, the predicted overall survival rate after diagnosis for acute leukemia was 21%. The adjusted hazard ratio for acute leukemia subtypes (aHR:4.9, 95% CI:2.3–10.4), history of relapse (aHR:3.9, 95% CI:1.0–7.9), participant age (aHR:1.25, 95% CI:1–1.75), hepatomegaly (aHR:2.7, 95% CI:1.36–5.36), and splenomegaly (aHR:2.29, 95% CI:1.2–4.4).

**Conclusion:**

The 5‐year overall survival rate was found to be 21%. The finding was remarkably lower than other published reports. Survival among acute leukemia patients was significantly associated with older age, history of relapse, hepatomegaly, splenomegaly, as well as certain subtypes. Therefore, improving early detection and initiation of treatment for all acute leukemia patients is necessary in order to improve patient's survival status.

## 1 BACKGROUND

The global burden of cancer is estimated to have risen to 19.3 million new cases, and 10 million deaths with 50 million of 5‐year prevalence in 2020.^1^ The American Cancer Society (ASCO) estimated that 13 800 cases of acute myelogenous leukemia (AML) and 6000 cases of acute lymphoblastic leukemia (ALL) were diagnosed in the United States.^2^ People with lower socioeconomic status are more likely to be exposed to cancer risk factors like smoking, obesity, and obstacles to effective cancer prevention, early diagnosis, and treatment. Lifestyle of the patients, genetic factors, therapeutic effects, and environmental factors have also been related to an elevated risk of cancer.^3^

Excluding non‐melanoma skin cancer, Leukemia was among the top 5 most common cancers, with 4361 new cases reported in Ethiopia in 2020. Leukemia incidence and death rates are predicted to be 46 and 39, respectively in Ethiopia in 2020.^4^ According to the cell types and the clinical course history Leukemia is classified into myeloid and lymphoid leukemia.^5^ Accordingly, ALL, chronic lymphocytic leukemia (CLL), AML and chronic myeloid leukemia (CML) are the four major types of leukemia (CML). Acute leukemia is very aggressive disorder that causes poor production of bone marrow cells that in turn leads to immunosuppression. Since acute leukemia has a very high complete remission rate (60%–67%) it requires rapid medical attention.^6^

According to studies, improvements in acute leukemia diagnosis, treatment choices, and patient risk classification instruments have enhanced survival rates.^7^ Data from surveillance, epidemiology and end results (SEER) showed that the 5‐year survival rate for patients with ALL increased from 31.6% in 1997–2002 to 39.0% in 2003–2008. However, compared to non‐Hispanic whites age‐adjusted 5‐year relative survival rates for African‐Americans and Hispanics remained lower.^6^ Cancer stage, sex, age, ethnicity, and socioeconomic position were also found to be determining factors of cancer survival. Black male adolescents and young adults with severe illness stages were shown to have worse survival rates.^8^ Although leukemia is becoming more prevalent and was a very serious problem in Ethiopia, it is not well studied about adult acute leukemia (AL) patients' survival rates. Thus, this study was designed to assess mortality risk factors among patients above 18 years with acute leukemia who were admitted to Tikur Anbessa Specialized Hospital (TASH) from January 2015 to December 2019.

## 2 MATERIALS AND METHODS

### 2.1 Study area, design, and period

A health institutional based retrospective cohort study was conducted from March to April, 2020.

Between January 2015 and December 2019, 203 medical records of adult patients with acute leukemia who were hospitalized to the hematology unit at Tikur Anbessa Specialized Hospital (TASH) were examined. Tikur Anbessa Specialized Hospital is the only public cancer care referral center in the country operating with a total of 700 beds. The hospital registry indicates that 370 000 to 400 000 cancer cases were monitored annually.

### 2.2 Definitions

The event in this study was death.

Acute leukemia: a patient with greater than or equal to 25% blasts in the BMA.

The time variable was time from diagnosis to the occurrence of death.

Participants with failure time or unknown outcome due to drops outs were considered as censored.

### 2.3 Populations

Between January 1, 2015 and December 31, 2019, any adult acute leukemia patients who were older than 18 and received care and therapy at Tikur Anbessa Specialized Hospital were enrolled.

#### 2.3.1 Eligibility

All patient records indicating an acute leukemia diagnosis with complete data records of (Patient characteristics: age, sex, marital status, residence, ethnicity, occupation, and religion were included. Acute leukemia, sub‐types such as AML, ALL, ALL‐B and immunophenotype‐T, hemoglobin level, white blood cells count phase of chemotherapy medical payment, remission, relapse place of relapse number of relapses, co morbidity co morbidity types splenomegaly, and hepatomegaly) were eligible, and those patient records lacking full information of the above variables were excluded.

#### 2.3.2 Sampling procedure

All patient records with acute leukemia diagnosis during the course of the investigation were included sequentially using a convenient sampling method.

### 2.4 Data collection tool

A structured data collection checklist was prepared to extract data from medical records and cancer registry books. In order to ensure the checklist's consistency to the study, it was pretested among 5% of the sample's total participants in a private clinic.

It includes an information about patient's sociodemographic variables including age, sex, marital status, ethnicity, country of residence, religion, and profession. It also included an information on the patient's acute leukemia including the date of diagnosis, length of hospitalization, type and phase of treatment, type of leukemia, a history of relapse, the location and number of relapses, co morbidity, hemoglobin, blood cell counts, and survey endpoints. In order to collect data, two experienced nurses with bachelor's degree were recruited and a daylong training was given.

### 2.5 Data processing and analysis

Data were coded recorded into an excel spread sheet, and then checked for consistency and completeness before being exported into the computer software Statistical Package for Social Sciences (SPSS) version 25.0 for analysis. Descriptive statistics such as frequency, median, mean and standard deviation were used to summarize participants characteristics.

The survival rates were calculated using the Kaplan–Meier curve technique. The log‐rank test was used to compare the groups' chances of surviving.

Bivariate and multivariate Cox proportional hazards regression models were done to identify the factors associated with survival status. Variables that show statistical significance in bivariate logistic regression analysis at (*p* < .25) were included into the multivariate Cox regression models to identify independent predictors for the patients' survival status and to estimate adjusted hazard ratios. Both crude and adjusted hazards ratio with 95% confidence interval were reported for variables that were statistically significant. Before the Cox proportional hazard regression model was applied, its multi‐collinearity was tested using the variance inflation factor (VIF) and pair‐wise correlation. To determine if the residuals satisfied the model test, Cox Snell residuals also utilized a goodness‐of‐fit test. The Schoenfeld residual statistical test was used to verify the proportional hazards assumption.

## 3 RESULTS

### 3.1 Socio‐demographic characteristics of the study participants

A total of 203 acute leukemia patients were observed from January 2015 to December 2019 in Tikur Anbessa Specialized Hospital (TASH), of which, 119 patient charts were found to have complete data and fulfilled eligibility criteria for this study. The mean age of the study participants was 40.5 ± 20.5 with minimum and maximum age range of 18–88 years old. Regarding to their religion about 53 (44.5%) were Orthodox Christian, 40 (33.6%) were Muslim, and 23 (19.3%) were protestants. More than half of study participants were from Oromia and Amhara regions (28.6% and 24.4%), respectively, and about half of the participants were rural inhabitants. Nearly half of them were married and 44 (37.0%) were farmers by occupation (Table 1).

**TABLE 1:** Socio‐demographic characteristics of acute leukemia patients in Tikur Anbessa Specialized Hospital from January2014to December2018, Ethiopia 2019 (*n* = 119).

Characteristics	Categories	Status at last contact		Total
		Death	Censored	
Age	18–34	13 (24.1%)	41 (75.9%)	54 (45.4%)
	35–64	16 (39%)	25 (61%)	41 (34.5%)
	≥65	17 (70.8%)	7 (29.2%)	24 (20.1%)
Sex	Male	26 (41.3%)	37 (58.7%)	63 (52.9%)
	Female	20 (35.7%)	36 (64.3%)	56 (47.1%)
Marital status	Married	23 (38.3%)	37 (61.7%)	60 (50.4%)
	Single	16 (34.8%)	30 (65.2%)	46 (38.7%)
	Divorced	7 (63.6%)	4 (36.4%)	11 (9.2%)
	Widowed	0	2 (100%)	2 (1.7%)
Residence	Rural	21 (35.0%)	39 (65.0%)	60 (50.4%)
	Urban	25 (42.4%)	34 (57.6%)	59 (49.6%)
Ethnicity	Amhara	16 (39.0%)	25 (61.0%)	41 (34.5%)
	Oromo	10 (30.3%)	23 (69.7%)	33 (27.7%)
	Tigre	12 (54.5%)	10 (45.5%)	22 (18.5%)
	Others	8 (34.8%)	15 (65.2%)	23 (19.3%)
Occupation	Farmer	25 (56.8%)	19 (43.3%)	44 (37.0%)
	Merchant	21 (48.8%)	22 (51.2%)	43 (36.1%)
	Civil servant	17 (62.9%)	10 (38.1%)	27 (22.7%)
	NGO	3 (0.6%)	2 (0.4%)	5 (4.2%)
Religion	Orthodox	32 (60.4%)	21 (39.6%)	53 (44.5%)
	Muslim	24 (60%)	16 (40%)	40 (33.6)
	Protestant	11 (47.8%)	12 (52.2%)	23 (19.3%)
	Catholic	2 (66.7%)	1 (33.3%)	3 (2.5%)
Region	Oromia	14 (41.2)	20 (58.8%)	34 (28.6%)
	Amhara	10 (34.5%)	19 (65.5%)	29 (24.4%)
	SNNPR	8 (38.1%)	13 (61.9%)	21 (17.6%)
	Tigray	9 (19.6%)	4 (5.5%)	13 (10.9%)
	Addis Ababa	4 (22.2%)	14 (19.2%)	18 (15.1%)
	Somalia	1 (25.0%)	3 (75.0%)	4 (3.4%)

### 3.2 Hematologic and pathologic characteristics

More than half of study participants were diagnosed with AML 62(52.1%) sub types, whereas B‐Immunophenotype were predominant groups 39 (68.4%) among ALL. The majority, research participant 93 (78.1%), of study participant did not have complete remission throughout survey period. Twenty‐seven (22.7%) of participants developed relapse during survey period, with the central nervous system 15 (55.6%) (Table 2).

**TABLE 2:** Hematologic and pathologic characteristics of acute leukemia patients at Tikur Anbessa Specialized Hospital, Addis Ababa, Ethiopia from January 2015 to December 2019 (*n* = 119).

Covariates	Categories	Status at last contact		Total
		Death	Censored	
Acute leukemia	AML	27 (43.5%)	35 (47.9%)	62 (52.1%)
	ALL	19 (33.3%)	38 (66.7%)	57 (47.9%)
ALL Sub‐types	Immunophenotype type‐ B	12 (30.8%)	27 (69.2%)	39 (68.4%)
	Immunophenotype type‐ T	7 (38.9%)	11 (61.1%)	18 (31.6%)
Hemoglobin	Abnormal	19 (34.5%)	36 (65.5%)	55 (46.2%)
	Normal	27 (42.2%)	37 (50.7%)	64 (53.8%)
White blood cells	≤10 000	0	26 (35.6%)	26 (21.8%)
	10 001‐49 999	13 (28.9%)	32 (43.8%)	45 (37.8%)
	≥50 000	33 (68.8%)	15 (20.5%)	48 (40.3%)
Phase of chemotherapy	Induction	18 (62.1%)	11 (37.9%)	29 (24.4%)
	Consolidation	18 (60.1%)	12 (40%)	30 (25.2%)
	Maintenance	21 (56.8%)	16 (43.2%)	37 (31.1%)
	Complete	12 (52.2%)	11 (47.8%)	23 (31.1%)
Aim of treatment	Radical	37 (40.7%)	54 (59.3%)	91 (76.5%)
	Palliative	9 (32.1%)	19 (67.9%)	28 (23.5%)
Medical payment	Self	30 (39.0%)	47 (61.0%)	77 (64.7%)
	Public	14 (35.9%)	25 (64.1%)	39 (32.8%)
	Others	2 (66.7%)	1 (33.3%)	3 (2.5%)
New cancer	Yes	7 (31.8%)	15 (68.2%)	22 (18.5)
	No	39 (40.2%)	58 (59.8%)	97 (81.5%)
Complete remission	Yes	7 (26.9%)	19 (76%)	26 (21%)
	No	40 (43%)	53 (57%)	93 (78.1%)
Splenomegaly	Yes	30 (50%)	30 (50%)	60 (50.4%)
	No	16 (27.1%)	43 (59.7%)	59 (49.6%)
Hepatomegaly	Yes	34 (64.2%)	19 (28%)	53 (44.5%)
	No	12 (18.2%)	54 (81.8%)	66 (55.5%)
Relapse	Yes	22 (81.5%)	5 (18.5%)	27 (22.7%)
	No	24 (26.1%)	68 (73.9%)	92 (77.3%)
Place of relapse	BM	4 (57.1%)	3 (42.9%)	7 (29.9%)
	CNS	14 (93.3%)	1 (6.7%)	15 (55.6%)
	BM and CNS	4 (80%)	1 (20)	5 (18.5%)
Number of relapses	1	6 (66.7%)	3 (33.3%)	9 (33.3%)
	2	9 (81.8%)	2 (18.2%)	11 (40.7%)
	≥3	7 (100%)	0	7 (25.9%)
Co morbidity	Yes	21 (75%)	7 (25%)	28 (23.5%)
	No	25 (27.5%)	66 (72.5%)	91 (76.5%)
Co morbidity types	Sepsis	10 (71.4%)	3 (28.6%)	13 (46.4%)
	Neutrophilic fever	11 (73.3%)	4 (26.7%)	15 (53.6%)
Death in 30 days	Yes	6 (53.8%)	0	6 (5%)
	No	40 (35.4%)	73 (64.6%)	113 (95%)

Abbreviations: ALL, acute lymphoblastic leukemia; AML, acute myeloid leukemia; BM, bone marrow; CNS, central nervous system.

### 3.3 Incidence and overall survival of acute leukemia patients during the follow‐up time

**FIGURE 1:**
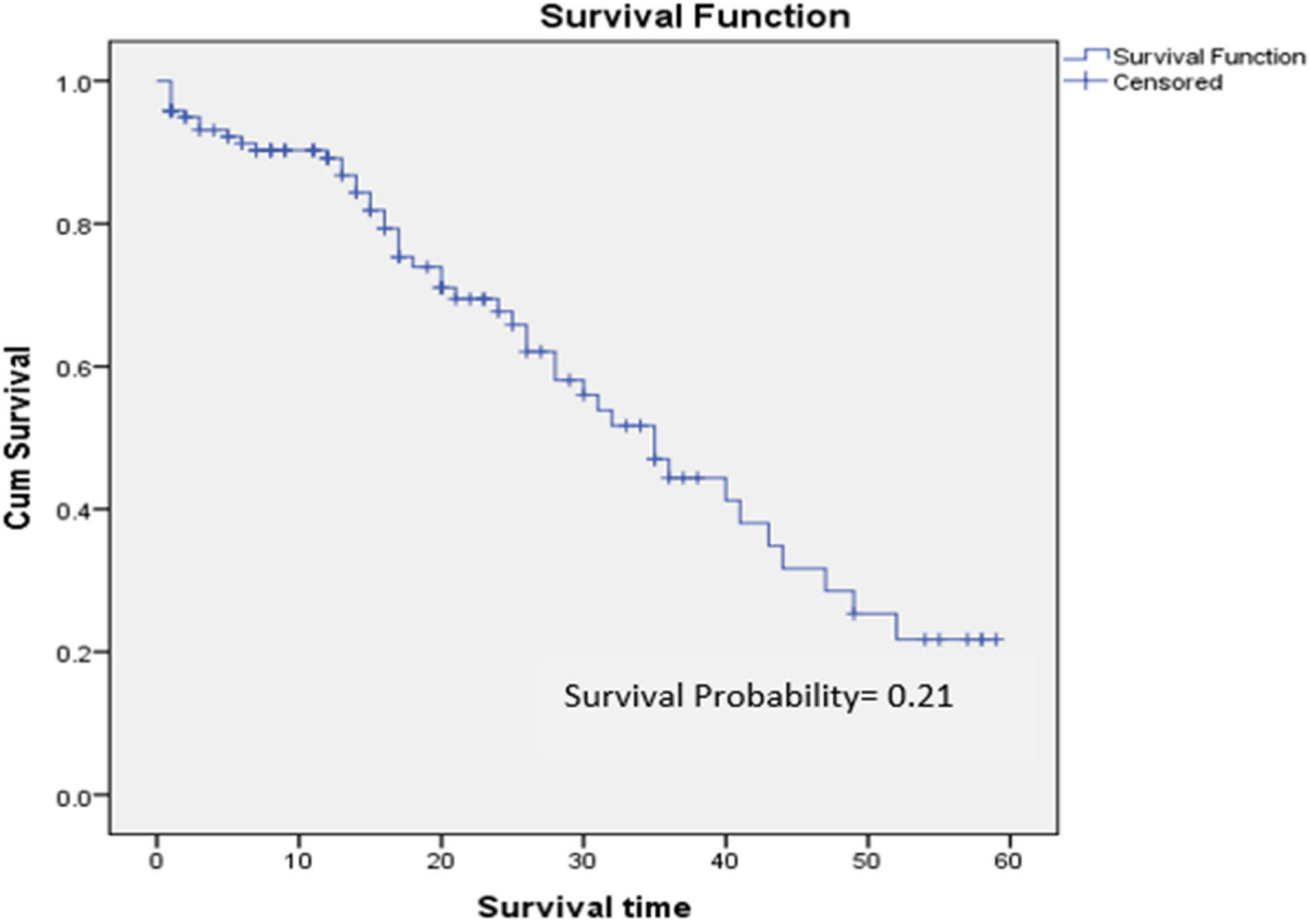
Overall survival probability of patients starting cancer chemotherapy in Tikur Anbessa Hospital from January 2015 to December 2019.

About 119 acute leukemia patients were followed for 60 months, yielding 196 person‐years‐time at risk. In the whole follow up period 46(38.7%) new deaths were observed resulting an overall death incidence rate of 23.5 (95% CL:18–52) per 100 person‐years. The median follow‐up period for this group is 17 (95% CI, 1–59) months, and the median survival was 35 months (95% CI, 28.3–41.7). In 60 months of follow‐up, the estimated survival rate for those with acute leukemia following therapy was 21%. At 12, 24, 36,48, and 60 months, the estimated cumulative survival probability was 89.1%, 67.7%, 44.4%, 28.5%, and 21%, respectively, (Figure 1).

As the follow‐up period lengthens, the likelihood of survival falls, which is a typical feature of survival data analysis. The KM survival curve makes it very evident that the period of the greatest mortality occurred between 12 and 36 months. As the follow‐up period lengthens, the likelihood of survival drops which is a typical feature of survival data analysis. The KM survival curve shows it very evident that the period of the greatest mortality occurred between 12 and 36 months.

### 3.4 Survival experience among different groups of leukemia patients in log‐rank test

**FIGURE 2:**
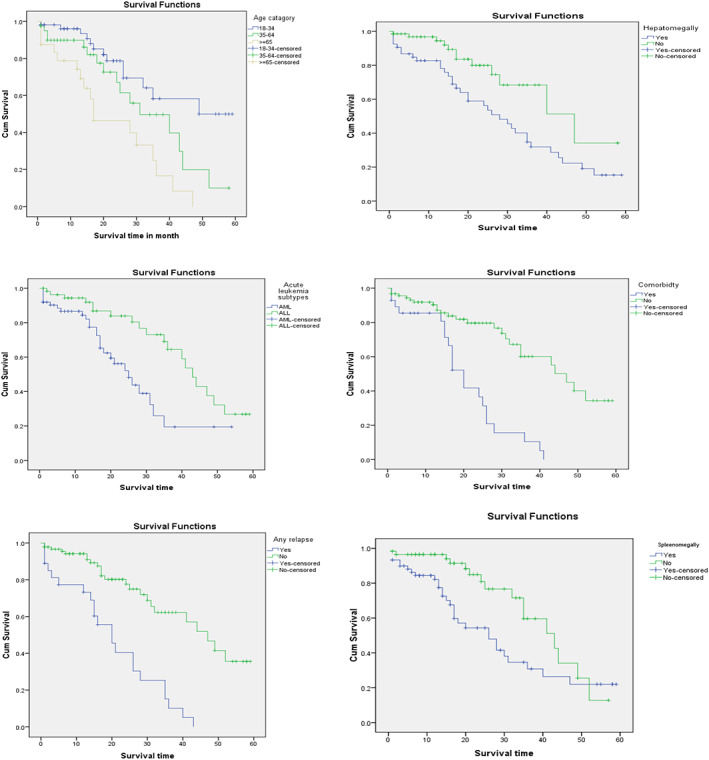
Survival outcomes (Kaplan–Meier analysis) by age of respondents (A), hepatomegaly (B), acute leukemia subtypes (C), comorbidity (D), relapse history (E), and splenomegaly (F) among acute leukemia patients treated in Tikur Anbessa Specialized Hospital, Addis Ababa, Ethiopia 2019 (*n* = 119).

The log‐rank test was performed to determine if there were any statistically significant variations in survival times among the various levels of the categorical factors taken into consideration in the study. There is a statistically significant difference in survival experience across categories of leukemia subtypes, age, co‐morbidity, relapse, splenomegaly, and hepatomegaly (Table 4 and Figure 2).

In accordance with the results of the Kaplan–Meier survival test (Table 4) patients with ALL subtypes (43 months) (95% CI:37.91–48.08) had a better survival time than those with AML subtypes AML 25(18.28–31.71).

Those without any co‐morbidities had a longer median survival period (47 months, 95% CI: 28.30–41.69) than those with co‐morbidities of any kind. The median survival times for patients with history of hepatomegaly and splenomegaly were 28 (95% CI:20–35.9) and 26 (95% CI:15.90–36.10) days, respectively, compared to those without a history of hepatomegaly and splenomegaly. Patients who did not experience relapse had a median survival time of 23 months. (95% CI:14.82–31.17), those with a history of leukemia relapse had a poorer median survival of 20 months (95% CI:13.21–26.78). Younger individuals (<34 months) had better survival than older individuals (95% CI:14.6–54) with a median survival of 44 months (Table 3 and Figure 2).

**TABLE 3:** Median survival time, 5‐year cumulative survival probability and log‐rank test according to socio demographic characteristics of leukemia patients in Tikur Anbessa Specialized Hospital, Ethiopia (*n* = 119).

Variables	Category	Median survival time, in months (95% CL)	Cumulative survival probability (%)	Log‐rank test	*p*‐value
Sex	Male Female	25 (22–35.9) 161 (6.7–23.3)	6.9 8.9	0.7	.4
Age	18–34 35–64 >64	42.9 (35.97–49.74) 32.9 (26.08–.39.75) 22.49 (15.86–29.11)	49.9 9.9 0.0	16.84	<.001
Marital status	Married Single	22 (14–25.8) 20 (13.3–30.7)	19.9 0.0	4.9	.18
Residence	Rural Urban	20 (16.6–23.4) 25 (19.5–30.5)	8.4 7.4	2.76	.26
Ethnicity	Amhara Oromo Tigre Others	22 (17–26.4) 20 (12.4–27.6) 34 (8.9–44) 21.5 (16.6–23.4)	9.4 13.4 19.1 8.7	10.6	.14
Occupation	Farmer Marchant Civil servant NGO	23 (9.4–36.6) 22 (10.4–32.6) 25 (12–37.9) 20 (9–37.9)	32.4 5.4 36.3 –	2.26	.52
Religion	Orthodox Muslim Protestant	34 (25–43) 23 (18–27.6) 16 (8–23.9)	6.4 2.3 9.3	7.8	.053
Region	Oromia Amhara SNNPR Addis Ababa Somalia	20 (4.9–39) 17 (8.7–25) 25 (5.9–44) 21 (95%: 25–43) 5 (95%: 5–22)	23 10.4 18.3 9.7 –	8	.16

**TABLE 4:** Median survival time, 5‐year cumulative survival probability and log‐rank test according to hematologic and pathologic characteristics of leukemia patients in Tikur Anbessa Specialized Hospital, Ethiopia (*n* = 119).

Variables	Category	Median survival time, in months (95% CL)	Cumulative survival probability (%)	Log‐rank test	*p*‐value
Acute leukemia types	AML ALL	25 (18.28–31.71) 43 (37.91–48.08**)**	19.4 27	9.27	<.05
ALL sub‐types	Immunophenotype type‐B Immunophenotype type‐T	34 (21.3–46.6) 20 (3.4–36.4)	7.6 0.0	1.07	.3
Hemoglobin	Normal Abnormal	22 (15–29) 23 (16–30.7)	17.6 5.7	0.03	.86
White blood cells	≤10 000 10 001–49 999	20 (13–26.7) 35 (22–48)	17.2 0.0	12	.07
Phase of chemotherapy	Induction Consolidation Maintenance Complete	23 (5.4–40.6) – 38 (34.4–81.6) 35 (20–46.1)	40.7 63.5 21.5 45.2	7.6	.054
Aim of treatment	Palliative Radical	58 (39.4–76.4) 34 (19–46.3)	4.8 19.1	0.67	.441
New cancer	Yes No	44 (33.4–46.4) 54 (38.4–69.4)	9.1 18.7	0.2	.6
Complete remission	Yes No	49.6 (39.4–76.4) 48 (29.4–55.4)	‐ 19.5	0.04	.85
Splenomegaly	Yes No	26 (15.90–36.10) 43 (31.32–54.67)	14.7 35.0	5.84	<.05
Hepatomegaly	Yes No	28 (20–35.9) 47 (27.7–60)	15.3 34.3	7.07	<.05
Relapse	Yes No	20 (13.21–26.78) 47 (37.28–56.72)	0.0 35.6	26.01	<.001
Number of relapses	1 2 ≥3	30 (16.57–33.42) 26 (15.57–29.42) –	7.1 0.0 0.0	1.71	.4
Comorbidity	Yes No	20 (16.57–23.42) 47 (28.30–41.69)	0.0 34.4	26.01	<.001
Comorbidity types	Sepsis Neutrophilic fever	25 (22.4–31.5) 30 (26.5–33)	7.8 7	0.37	.8
Death in 30 days	Yes No	– 3 3(27.2–47.4)	0.0 16.8	0.19	.66

**TABLE 5:** Cox regression analysis of acute leukemia patients on follow‐up at Tikur Anbessa Specialized Hospital, Addis Ababa, Ethiopia, 2019 (*n* = 119).

Variables	Categories	Censored	cHR	(95%CI)	aHR	(95% CI)	*p*‐value
Leukemia type	AML	27 (43.5%)	35 (56.5%)	1.36–4.68	4.9	2.3–10.4	.00*
	ALL	19 (33.9%)	37 (66.1%)				
Relapse	Yes	22 (81.5%)	5 (18.5%	2.3–8.8	3.9	1.0–7.9	.002*
	No	24 (26.1%)	68 (73.9%)				
Co‐morbidity	Yes	21 (75%)	7 (25%)	2.34–8.1	1.6	0.8–3.3	.18
	No	25 (75%)	66 (72.5%)				
Age	18–34	13 (24.1%)	41 (75.9%)				
	35–64	16 (39.0%)	25 (61.0%)	0.24–0.95	0.6	0.3–1.4	.25.
	≥65	17 (70.8%)	7 (29.2%)	0.12–0.50	1.25	1.3–1.75	.01*
Splenomegaly	Yes	30 (50%)	30 (50%)	1.13–3.83	2.29	1.2–4.4	.01*
	No	16 (27%)	43 (73%)				
Hepatomegaly	Yes	34 (64.2%)	19 (28%)	1.2–4.6	2.7	1.36–5.36	.004*
	No	12 (18.2%)	54 (81.8)				

*Note*: Bivariate<0.25*; multivariate<0.05*.

### 3.5 Bivariate and multivariate Cox‐regression analysis of acute leukemia patients on follow‐up at Tikur Anbessa Specialized Hospital, Addis Ababa, Ethiopia, 2019 (*n* = 119)

Multivariate Cox proportional hazards analysis revealed that acute leukemia sub types (aHR:4.9.95% CI:2.3–10.4), history of relapse (aHR:3.9, 95% CI:1.0–7.9), advanced age (aHR:1.25, 95% CI:1.3–1.75), splenomegaly (aHR:2.29, 95% CI:1.2–4.4), and hepatomegaly (aHR:2.7.95% CI:1.36–5.36) were independent prognostic indicators for better survival in terms of overall survival acute leukemia (Table 5).

## 4 DISCUSSION

Recent progress in understanding survival and predictors of mortality has arouse growing interest in the epidemiology of hematologic malignancies. In this study, we evaluated survival patterns and predictors of outcomes in acute leukemia patients in Ethiopia. In the present retrospective cohort study, the 1‐, 2‐, 3‐, 4‐, and 5‐year survival rate of acute leukemia was respectively 89.1%, 67.7%, 44.4%, 28.5%, and 21%, respectively. The results indicated that Advanced age, history of relapse, hepatomegaly, splenomegaly, and leukemia subtype had a significant effect on survival rate. Overall acute leukemia survival rate was high in developed world, while being very low in developing countries indicating that leukemia treatment centers were relatively young in these nations.^9^

On the other hand, there are not many local studies available for the comparison of acute leukemia survival rates. In this study, the survival rate for a patient with acute leukemia was 21%. This was lower than the rates reported for other developing countries, which was 86% for India^10^ and 52% for Nigeria.^11^ The variation might be due to the fact that there is only one leukemia treatment facility in the country, in which may lack the patients in the present study location from receiving early treatment. In this study, AML is the deadliest form of acute leukemia, and its survival is substantially lower than that of ALL subtypes. The overall survival rate for AML was (19%), which is nearly similar to the study in the USA that showed 18.2%, however, the present study for ALL found 27%, which was lower than USA 44.0%.^12^ Studies conducted in German showed 5‐year survival probabilities for AML was 44.3%^13^ and for ALL was 43.4%^14^ subtype which is higher than current study for both types.

Overall 5‐year survival was estimated at 43.4% for Germany which is higher than the current finding, this might be due to scarcity of Leukemia management centers, lack of skilled human power in the cancer center or it may be due to SES, genetic and geographic variations in study cohort.^15^ In contrast to the Swedish study, which found no difference in survival across subtypes, the current study found a substantial difference in survival between the AML and ALL subtypes, which are 19.4% and 27%, respectively,^16^ this could be there is poor treatment center for leukemia in Ethiopia specially AML subtypes, that may result very poor survival of AML in Ethiopia or it may be due to socio‐economic, genetic or geographical variation. Our 5‐year acute leukemia survival rate was lower than the survival rate reported on patients of USA in both ALL and AML subtypes.^17^ The current study found a higher value in induction death (13%) than the US found 5.9% ± 1.9%^18^ induction deaths, which is suggestive of a different regimen or low cytotoxicity management. Furthermore, the current study's complete remission rate was very low (26%), compared to the 57% of the American study.^19^

In comparison to the Canadian study, which covers patients with ALL and AML, both types of acute leukemia had lower overall survival rates (64% and 62%), respectively.^7^ This is likely because there is inadequate or no medical care for an advanced tumor in the existing setup. In the current study, leukemia subtype, history of relapse, hepatomegaly, splenomegaly, and age were the main predictors of acute leukemia mortality in Tikur Anbessa Specialized Hospital.

According to the present study findings, the survival rate of AML (19%) subtypes were lower survival rates than ALL (27%) subtypes, which is consistent with the report from the American cancer association that showed AML (66%) subtypes have lower survival rates than ALL (90%) subtypes.^9^ Another study done in Nigeria also supported the current finding that ALL (77.7%) had better survival than AML (44%) subtypes.^20^ Although the WBC count and survival of leukemia patients were not significantly correlated in this investigation, a study carried out in the USA suggested that a higher WBC count (≥50 000/μL) was associated with worse outcomes.^21^ Our study found that the history of relapses was the primary predictor acute leukemia survival, and those who have relapse history were four times more likely than those of the controls which is similar to the previous study in British^21^ and Germany.^22^

Current finding shows that, the risk of mortality increased by 1.25 times as people aged (95%CI: 1.3, 1.75) which is consistent with research carried out in Sweden and the United Kingdom^23^, ^24^ This result also inline Netherlands showed older age has a lower survival for acute leukemia patients.^25^ The findings of the current study demonstrated that an individual with acute leukemia who are older than 60 had worse treatment outcomes and survival rates. This is consistent with research of a similar nature conducted in elsewhere.^15^ Age at diagnosis was one of the risk factors that affected leukemia patients' chances of surviving. Leukemia patients over 65 were shown to have a higher mortality risk than those under 34, which was supported by UK research that found those under 34 had a better outlook.^26^ According to data from other research, the 5‐year survival of acute leukemia patients approaches 40% in younger patients and 15% in older individuals.^14^

This study also showed that worsened survival among age group >64 years which is similar with previous finding increased age have (>64) have a worse survival.^27^ The finding was also supported by the Canadian study which showed that age at diagnosis is linked with the survival of acute leukemia cases in which older ages are at a higher risk for death in comparison to the young ones.^23^ ASCO's finding reports that age > 40 years 2.2 times hazards to death than (≤40) years^24^ the current finding is also in line with it in which older^26^ age (>64) was 1.25 hazards of death in comparison with age < 34 years. Similar to current finding, another US based study showed that increased age has significant impacts on survival in consecutive age groups above 45–54 years in all leukemia subtypes have lower survival.^12^

According to the US study, leukemia subtype and sex are the key determinant factor of survival in leukemia patients. Hence, the study shown that the female patients with a leukemia diagnosis had a better survival rate than male patients.^12^ There was no correlation between sex and survival status among acute leukemia cases in the current investigation, despite the fact that the study's previous findings indicated that there were survival differences between two genders this may be due to methodological or genetic variation in white and race cohorts.

This study had some limitations. Firstly, we could not determine the impact of chemotherapy and immune‐therapy on survival because we lack full course of treatment in most patient's chart. Secondly, since the sample size were small it lacks generality. The study was retrospective with a possibility of bias from inaccurate survival and predictors information due to the missing data or inadequate reporting by hospital. We do not reported hospital stay due to we have no clear data on patient stay at hospital. Cause‐specific survival was not determined due to lack of data on specific cause of death, this may overestimate acute leukemia‐related mortality rate.

## 5 CONCLUSION AND RECOMMENDATIONS

The survival rate for individuals with acute leukemia cases was very low. Advanced age, history of relapse, hepatomegaly, splenomegaly, and leukemia subtype were explained to be reliable predictors of death in patients with acute leukemia. Therefore, improving early detection and initiation of treatment for all acute leukemia patients is necessary in order to improve the patient's survival status. Further large‐scale prospective follow‐up studies could be recommended on specific types of acute leukemia.

## AUTHOR CONTRIBUTIONS

**Bargude Balta:** Conceptualization (equal); data curation (equal); formal analysis (equal); investigation (equal); methodology (equal); writing – original draft (equal); writing – review and editing (equal). **Tigistu Gebreyohannis:** Software (equal); supervision (equal); writing – review and editing (equal). **Erdaw Tachbele:** Conceptualization (equal); data curation (equal); software (equal); supervision (equal); validation (equal); visualization (equal); writing – original draft (equal); writing – review and editing (equal).

## CONFLICT OF INTEREST STATEMENT

We read the journal's policy and the authors of this manuscript have no conflicts of interest.

## ETHICS STATEMENT

The official clearance later (Ref No. NUR.128/19) was obtained from research and ethics committee (REC) of School of Nursing and Midwifery, College of Health Sciences, Addis Ababa University. Permission to access the patients' records was granted from hospital officials Patient information was anonymous and kept confidential. As we are reporting a retrospective study of medical records, requirement for informed consent was not required.

## Data Availability

All‐important data were available with correspondent authors and shared accordingly reasonable requests

## References

[1] Sung H , Ferlay J , Siegel RL , et al. Global cancer statistics 2020 : GLOBOCAN estimates of incidence and mortality worldwide for 36 cancers in 185 countries. CA Cancer J. 2021;71:1‐41. 10.3322/caac.21660 33538338

[2] Rose‐Inman H , Kuehl D . Acute leukemia. Hematol Oncol Clin North Am. 2017;31(6):1011‐1028. doi:10.1016/j.hoc.2017.08.006 29078921

[3] Siegel RL , Miller KD , Jemal A . Cancer statistics, 2019. CA Cancer J Clin. 2019;69(1):7‐34. 30620402 10.3322/caac.21551

[4] Sharma R , Aashima NM , Fronterre C , et al. Mapping cancer in Africa: a comprehensive and comparable characterization of 34 cancer types using estimates from GLOBOCAN 2020. Front Public Heal. 2022;10(4):1‐14. 10.3389/fpubh.2022.839835 PMC9082420 35548083

[5] Parvareh M , Khanjani N , Farahmandinia Z , Nouri B . Original Article. Iran J Heal Sci. 2015;3(4):24‐32.

[6] Pulte D , Redanie MT , Jansen L , Brenner H , Jeffreys M . Recent trends in survival of adult patients with acute leukemia: overall improvements, but persistent and partly increasing disparity in survival of patients from minority groups. Haematologica. 2013;98(2):222‐229. 22929974 10.3324/haematol.2012.063602 PMC3561429

[7] Kraguljac AP , Croucher D , Christian M , et al. Outcomes and predictors of mortality for patients with acute leukemia admitted to the intensive care unit. Hindawi. 2016;2016(8):1‐7. 10.1155/2016/3027656 PMC4944052 27445524

[8] Moke DJ , Tsai K , Hamilton AS , et al. Emerging cancer survival trends, disparities, and priorities in adolescents and young adults: a California cancer registry‐based study. JNCI Cancer Spectr. 2019;3(2):1‐9. 10.1093/jncics/pkz031 PMC6597054 31276099

[9] Muzamil J . Acute lymphoblastic leukemia, the Indian scenario. MOJ Cell Sci Rep. 2018;5(2):1‐6.

[10] Kulkarni KP , Arora RS , Marwaha RK . Survival outcome of childhood acute lymphoblastic leukemia in India. J Pediatr Hematol Oncol. 2011;33(6):475‐479. 21792045 10.1097/MPH.0b013e31820e7361

[11] Egesie OJ , Agaba PA , Silas OA , et al. Presentation and survival in patients with hematologic malignancies in Jos, Nigeria: a retrospective cohort analysis. J Med Trop. 2018;20(1):116‐122. 10.4103/jomt.jomt_8_18 PMC6024253 29963503

[12] Gardin C , Turlure P , Fagot T , et al. Postremission treatment of elderly patients with acute myeloid leukemia in first complete remission after intensive induction chemotherapy: results of the multicenter randomized acute leukemia French association (ALFA) 9803 trial. Blood. 2007;109(12):5129‐5135. 17341661 10.1182/blood-2007-02-069666

[13] Büchner T , Schlenk RF , Schaich M , et al. Acute myeloid leukemia (AML): different treatment strategies versus a common standard arm‐combined prospective analysis by the German AML intergroup. J Clin Oncol. 2012;30(29):3604‐3610. 22965967 10.1200/JCO.2012.42.2907

[14] Pulte D , Jansen L , Gondos A , et al. Survival of adults with acute lymphoblastic leukemia in Germany and the United States. PloS One. 2014;9(1):e85554. 24475044 10.1371/journal.pone.0085554 PMC3903479

[15] Juliusson G , Karlsson K , Lazarevic VL , et al. Hematopoietic stem cell transplantation rates and long‐term survival in acute myeloid and lymphoblastic leukemia: real‐world population‐based data from the Swedish acute leukemia registry 1997‐2006. Cancer. 2011;117(18):4238‐4246. 21387283 10.1002/cncr.26033

[16] Chevallier P , Delaunay J , Turlure P , et al. Long‐term disease‐free survival after gemtuzumab, intermediate‐dose cytarabine, and mitoxantrone in patients with CD33+ primary resistant or relapsed acute myeloid leukemia. J Clin Oncol. 2008;26(32):5192‐5197. 18854573 10.1200/JCO.2007.15.9764

[17] Dores GM , Devesa SS , Curtis RE , Linet MS , Morton LM . Acute leukemia incidence and patient survival among children and adults in the United States, 2001–2007. Blood. 2012;119(1):34‐43. 22086414 10.1182/blood-2011-04-347872 PMC3251235

[18] Gupta S , Pole JD , Baxter NN , et al. The effect of adopting pediatric protocols in adolescents and young adults with acute lymphoblastic leukemia in pediatric vs adult centers: an IMPACT cohort study. Cancer Med. 2019;8(5):2095‐2103. 30912628 10.1002/cam4.2096 PMC6536996

[19] Hunger SP , Lu X , Devidas M , et al. Improved survival for children and adolescents with acute lymphoblastic leukemia between 1990 and 2005: a report from the children's oncology group. J Clin Oncol. 2012;30(14):1663‐1669. 22412151 10.1200/JCO.2011.37.8018 PMC3383113

[20] Bailey C , Richardson LC , Allemani C , et al. Adult leukemia survival trends in the United States by subtype: a population‐based registry study of 370,994 patients diagnosed during 1995–2009. Cancer. 2018;124(19):3856‐3867. 30343495 10.1002/cncr.31674 PMC6392057

[21] Marks DI , Paietta EM , Moorman AV , et al. T‐cell acute lymphoblastic leukemia in adults: clinical features, immunophenotype, cytogenetics, and outcome from the large randomized prospective trial (UKALL XII/ECOG 2993). Blood. 2009;114(25):5136‐5145. 19828704 10.1182/blood-2009-08-231217 PMC2792210

[22] Bejanyan N , Weisdorf DJ , Logan BR , et al. Survival of patients with acute myeloid leukemia relapsing after allogeneic hematopoietic cell transplantation: a Center for International Blood and Marrow Transplant Research Study. Biol Blood Marrow Transplant. 2015;21(3):454‐459. doi:10.1016/j.bbmt.2014.11.007 25460355 PMC4329076

[23] Short NJ , Zhou S , Fu C , et al. Association of measurable residual disease with survival outcomes in patients with acute myeloid leukemia: a systematic review and meta‐analysis. JAMA Oncol. 2020;6(12):1890‐1899. 33030517 10.1001/jamaoncol.2020.4600 PMC7545346

[24] Juliusson G , Antunovic P , Derolf Å , et al. Age and acute myeloid leukemia: real world data on decision to treat and outcomes from the Swedish acute leukemia registry. Blood. 2009;113(18):4179‐4187. 19008455 10.1182/blood-2008-07-172007

[25] Dinmohamed AG , Szabó A , Van Der Mark M , et al. Improved survival in adult patients with acute lymphoblastic leukemia in The Netherlands: a population‐based study on treatment, trial participation and survival. Leukemia. 2016;30(2):310‐317. 26286115 10.1038/leu.2015.230

[26] Hoelzer D , Gökbuget N , Digel W , et al. Outcome of adult patients with T‐lymphoblastic lymphoma treated according to protocols for acute lymphoblastic leukemia. Blood. 2002;99(12):4379‐4385. 12036865 10.1182/blood-2002-01-0110

[27] Ellison LF . Increasing survival from leukemia among adolescents and adults in Canada: a closer look. Heal Reports. 2016;27(7):19‐26. 27439000

